# Human F_1_F_0_ ATP Synthase, Mitochondrial Ultrastructure and OXPHOS Impairment: A (Super-)Complex Matter?

**DOI:** 10.1371/journal.pone.0075429

**Published:** 2013-10-02

**Authors:** Johann Habersetzer, Isabelle Larrieu, Muriel Priault, Bénédicte Salin, Rodrigue Rossignol, Daniel Brèthes, Patrick Paumard

**Affiliations:** 1 Laboratoire des Systèmes Transducteurs d′Energie et Morphologie Mitochondriale, Université Bordeaux Segalen, IBGC, UMR 5095, Bordeaux, France; 2 CNRS, IBGC, UMR 5095, Bordeaux, France; 3 MRGM, Laboratoire Maladies Rares: Génétique et Métabolisme, Université Bordeaux Segalen, Bordeaux, France; University of Saskatchewan, Canada

## Abstract

Mitochondrial morphogenesis is a key process of cell physiology. It is essential for the proper function of this double membrane-delimited organelle, as it ensures the packing of the inner membrane in a very ordered pattern called *cristae*. In yeast, the mitochondrial ATP synthase is able to form dimers that can assemble into oligomers. Two subunits (*e* and *g*) are involved in this supramolecular organization. Deletion of the genes encoding these subunits has no effect on the ATP synthase monomer assembly or activity and only affects its dimerization and oligomerization. Concomitantly, the absence of subunits *e* and *g* and thus, of ATP synthase supercomplexes, promotes the modification of mitochondrial ultrastructure suggesting that ATP synthase oligomerization is involved in *cristae* morphogenesis. We report here that in mammalian cells in culture, the shRNA-mediated down-regulation of subunits *e* and *g* affects the stability of ATP synthase and results in a 50% decrease of the available functional enzyme. Comparable to what was shown in yeast, when subunits *e* and *g* expression are repressed, ATP synthase dimers and oligomers are less abundant when assayed by native electrophoresis. Unexpectedly, mammalian ATP synthase dimerization/oligomerization impairment has functional consequences on the respiratory chain leading to a decrease in OXPHOS activity. Finally these structural and functional alterations of the ATP synthase have a strong impact on the organelle itself leading to the fission of the mitochondrial network and the disorganization of mitochondrial ultrastructure. Unlike what was shown in yeast, the impairment of the ATP synthase oligomerization process drastically affects mitochondrial ATP production. Thus we propose that mutations or deletions of genes encoding subunits *e* and *g* may have physiopathological implications.

## Introduction

Mitochondria host various functions and are at the center of eukaryotic cells metabolism. These organelles produce anabolic pathways precursors. They also achieve the ultimate steps of the oxidative catabolism responsible for the conversion of nutrients into the molecular energy currency ATP. This central role explains the growing interest in mitochondria as these organelles appear to be involved in a broad range of human pathologies (see [1], [2] for review). Mitochondria harbor in their inner membrane the protein complexes involved in the oxidative phosphorylation process (OXPHOS) which is constituted by the four complexes of the respiratory chain, the F_1_F_0_ ATP synthase (or ATP synthase) and both the ATP/ADP carrier and Pi/H^+^ carrier. The ATP synthase complex is responsible for the production of the majority of cellular ATP under aerobic conditions.

The overall organization of ATP synthase is conserved from yeast to mammalian cells [3] This 600 kDa enzyme is constituted by two sectors: *(i)* the soluble sector named F_1_ that protrudes into the mitochondrial matrix space and bears the catalytic activity, is composed of five subunits in all eukaryotic cells (subunits *α*, *β*, *γ*, *δ* and *ε*); *(ii)* the membranous sector named F_0_, that ensures the proper anchorage and stabilization of the F_1_ moiety, forms a specific proton-conducting pathway and is composed of a core of seven subunits (subunits *b*, *F_6_*, *d*, *f*, *a*, *A6L* and *OSCP*) plus organism specific subunits (subunits *i* and *k* in *S. cerevisiae*, subunit *s* in *B. taurus*) and two dimerization subunits (subunits *e* and *g*). When the two sectors are coupled, the enzyme functions as a H^+^-transporting ATP synthase which uses the electrochemical proton gradient generated by the respiratory chain to synthesize ATP from ADP and inorganic phosphate [4], [5].

Using Blue Native PolyAcrylamide Gel Electrophoresis (BN-PAGE), it has been shown that non-ionic detergents like digitonin allow extraction of F_1_F_0_ ATP synthase dimers and oligomers of dimers from both yeast and mammalian mitochondrial inner membrane [6]–[9]. *In vivo* ATP synthase oligomerization has been confirmed by direct electron microscopy observations. Indeed, the very first experiments were done in *Paramecium multimicronuclatum*, using freeze-fracture and deep-etching, revealing the presence of double rows of F_1_ sized particles along the edge of tubular *cristae* [10]. What was first thought to be somehow peculiar of paramecia was later reported in mitochondria from other species including mammalian cells [11]. It is now well accepted that ATP synthase complexes are organized as dimer ribbons along highly curved *cristae* membranes as shown by cryoelectron tomography [12], [13].

In the yeast *S. cerevisiae*, subunits *e* and *g* are involved in the dimerization and the oligomerization processes of ATP synthase. Whenever either one of the genes encoding these proteins is deleted, although the enzyme is fully functional, mitochondrial ATP synthase dimers and oligomers are no longer detectable by BN-PAGE [6], [8]. Mitochondria from these cells present structural alterations where classical *cristae* are no longer visible and replaced by onion like structures and large digitations surrounding other organelles (see [14] for review). The observation of mitochondrial morphology upon modulation of the amount of subunits *e* and *g* has revealed a correlation between the oligomeric organization of ATP synthase and mitochondrial morphology [15]. As hypothesized by Allen, association of dimeric ATP synthases to form oligomers could very well be responsible for the curvature of the inner mitochondrial membrane, leading to the formation of *cristae* [16].

Although the ability of ATP synthase to form oligomers is conserved in mammals, most studies investigating the mechanisms involved in this process were performed in yeast. Oligomer formation requires the presence of two interfaces [17]. It has been proposed that the first interface is composed of subunits of the peripheral stalk (yeast subunits *4* and *h*: homologues of mammalian subunits *b* and *F6* respectively). Because these subunits are essential for the correct assembly of the enzyme, the interactions involved in this interface cannot be characterized. The second interface was extensively studied. It involves the membranous dimerization motif GXXXG of subunits *e* and *g* and the first membrane spanning segment of subunit *4* [18]–[20]. To date, the involvement of subunits *e*, *g*, *F6* or *b* in the formation of mammalian ATP synthase supercomplexes (dimers and oligomers) has not been studied. The natural inhibitor of the sector F_1_ (*IF1*) has been proposed to participate in the stabilization of the mammalian ATP synthase dimer [21], [22]. Since the yeast ATP synthase dimer formation does not require the homologue of *IF1* (*Inh1*) [23], its involvement in the oligomerization process is still a matter of debate [24]. Consequently, despite the fact that ATP synthase oligomerization is conserved from yeast to mammals, there might be some subtle differences in the way that the subunits identified in yeast interact with each other to promote the formation of these supercomplexes in mammals.

Since the late fifties, a growing number of evidence of the implication of mitochondria in various pathologies was obtained. Mitochondrial dysfunctions and more particularly mutations in genes encoding specific ATP synthase subunits lead to various syndromes [25]. Among them, NARP (Neuropathy, Ataxia and Retinitis Pigmentosa) and MILS (Maternally Inherited Leigh Syndrom) are representative for the symptoms of patients suffering from these pathologies (lactic acidosis, myopathy, cardiomyopathy, mental retardation, dementia, blindness). Associated with these symptoms, there is often a strong alteration in mitochondrial shape and size, but it is still unknown whether these phenotypical modifications are a cause or a consequence of these pathologies. In some cases, morphologies characterized by concentric multilamellar *cristae* are observed which resemble those seen after the disruption of ATP synthase oligomerization in yeast [26], [27]. To date, in these pathologies, it has not been established whether mitochondrial ultrastructure alteration could be the consequence of ATP synthase oligomerization impairment.

This last decade leaves us with open questions regarding the extrapolation of the data obtained in yeast to mammalian cells. For instance, we still do not know if the molecular mechanisms of ATP synthase oligomerization are conserved between these species. It is also unknown if the relationship between *cristae* morphogenesis impairment and ATP synthase oligomerization is conserved in mammalian cells and if this phenomenon is involved in mitochondrial pathologies.

In this work we addressed these questions by attenuating the expression of human subunits *e* and *g* homologues (encoded by *ATP5I* and *ATP5L* genes respectively) in HeLa cells. We demonstrate that human subunits *e* and *g* are involved in the stabilization of ATP synthase monomer and supercomplexes (dimer and oligomer). Surprisingly, the down-regulation of these subunits affects cell physiology and the mitochondrial network. These findings may be of great interest considering that 80% of mitochondrial diseases are from unknown origin. Thus, it is very tempting to speculate that the impairment of ATP synthase oligomerization may have physiopathological implications.

## Materials and Methods

### Cell Lines and Culture

HEK 293T cells (CRL-11268) and HeLa cells (CCL_2) used in this study are from the Global Bioresource Center (ATCC).

All cell culture materials were obtained from Gibco (Invitrogen). HEK and HeLa cells were grown respectively in DMEM and RPMI (4.5 g/L glucose). All growth media were supplemented with 10% glutamax (from Gibco Invitrogen), penicillin (100 U/mL), streptomycin (100 mg/mL) and 10% fetal calf serum (PAA Fetal Serum Standard Quality (R)).

### Plasmid Construction

pLB-IL was obtained after substitution of the enhanced green fluorescent protein (E-GFP) gene in pLB (Addgene plasmid 11619, [28]) by a GFP gene that is mitochondrial-targeted thanks to the Cox8 import sequence (Mt-GFP). Mt-GFP gene was amplified by PCR with oligonucleotides 5′-GGG GAC CGG TAA TTC GTG CCA TCA TGT CC- 3′ and 5′- GGG GGA ATT CGG ATC CTC AGT TGT ACA GTT C- 3′ using the plasmid pMtGFP quantum as matrix [29]. The fragment amplified was inserted in the pLB plasmid using restriction sites *Age*I/*Eco*RI.

### HIV-1 Lentivirus-based Vectors Construction

HIV-1 lentivirus-based vectors were used to introduce shRNAs into HeLa cells. The DNA sequences encoding shRNAs were cloned under the control of the U6 promoter between *Hpa*I and *Xho*I sites in pLB-IL. HEK 293T cells were used as packaging cells, and virus production matrix. Briefly, cells were co-transfected with pLB-IL, psPAX2 (Addgene plasmid 12260) and pLP-VSVG (Invitrogen). 48 hours after transfection, lentiviral particles were separated from the culture medium by ultracentrifugation (1 h30 at 120000*×g* at 4°C) on a 20% sucrose cushion and resuspended in PBS. After titration, lentiviral particles were frozen and kept at −80°C. Sequences designed for subunits *e* and *g* interference were respectively: 5′-GAA TAA AGC TTC CTG TGT T-3′ (nucleotides 334 to 352 of *ATP5I*; accession no. NM_007100.2) and 5′-GTC AAT AGT GCT CAG ACT G-3′ (nucleotides 446 to 464 of *ATP5L*; accession number NM_006476). To control the potential side effects of transducing cells with lentiviral particles, a control experiment was systematically run in parallel with lentiviral particles where a sequence (5′-ACT ACC GTT GTT ATA GGT G-3′) that does not target any protein encoding mRNA of the cell is encapsulated. This control is referred to as *Scramble*. HeLa cells were transduced by incubating growing cultures with the lentiviral particles for 72 hours (MOI = 3 and MOI = 4 for *ShATP5I* and *ShATP5L* lentiviral particles respectively).

### Total Protein Extraction

Cells were scraped and washed twice in Dulbecco phosphate buffer saline (DPBS) by centrifugation for 2 min at 200×*g*. Proteins were precipitated by addition of trichloroacetic acid (TCA) 1.5 M and incubated during 20 min at −20°C. After centrifugation for 3 min at 12000*×g*, TCA was removed and the pellet was rinsed with acetone. Finally, the pellet was solubilized with 0.1 M HEPES, 5% SDS (weight/volume, w/v), pH 7.55.

### Mitochondria Enrichment

Mitochondria enrichment was accomplished as previously described [30]. Briefly, after 9 days of amplification, 3×10^7^ cells were scraped and washed twice in DPBS by centrifugation for 15 min at 200×*g* at 4°C. The pellet was then solubilized in 6 mL of MB buffer (210 mM mannitol, 70 mM sucrose, 1 mM EDTA, 10 mM HEPES pH 7.2–7.4, protease inhibitor). Cells were broken by strokes of a tight-fitting Dounce homogenizer and mitochondrial fractions were isolated by two differential centrifugations (5 min at 1000*×g* at 4°C and 10 min at 12000*×g* at 4°C) and resuspended in 400 µL of MB buffer.

### BN/CN-PAGE

500 µg of mitochondrial proteins were pelleted by centrifugation for 10 min at 12000×*g* at 4°C. The pellet was solubilized at 10 mg/mL in extraction buffer (150 mM potassium acetate, 12% glycerol (w/v), 2 mM 6-aminocaproic acid, 1 mM disodic EDTA, 30 mM HEPES pH 7.4) supplemented with different digitonin concentrations (from 1.5 to 5% (w/v)). After 30 min on ice, mitochondrial detergent extracts were centrifuged for 30 min at 24000×*g* at 4°C. Supernatants were supplemented with 36 mM 6-aminocaproic acid for clear-native polyacrylamide gel electrophoresis (CN-PAGE) or 36 mM 6-aminocaproic acid, 0.25% (w/v) Serva Blue G for blue-native polyacrylamide gel electrophoresis (BN-PAGE) and loaded onto polyacrylamide gradient (3–13%) gels.

BN-PAGE experiments were carried out as previously described [31] and CN-PAGE experiments were carried out as described by Wittig [32] except that polyacrylamide gradient was supplemented with 0.025% (w/v) of digitonin.

### In-gel ATPase Activity

After electrophoresis the gel was incubated with 4 mM ATP, 5 mM MgCl_2_, 0.05% (w/v) lead acetate, 0.05% (w/v) Triton X-100 and 50 mM glycine-NaOH pH 8.4. ATPase activity was revealed by a white precipitate of lead phosphate [33]. If necessary, this revelation was enhanced with an ammonium sulfide treatment [34].

### SDS-PAGE and Western Blot Analyses

Sodium dodecyl sulfate polyacrylamide gel electrophoresis (SDS-PAGE) were done using Tris-tricine 16.5% polyacrylamide slab gels according to Schägger and von Jagow [35] or Tris-glycine 12% polyacrylamide as described by Laemmli [36]. For western-blotting, proteins were electro-transferred onto nitrocellulose membranes (Membrane Protean BA83, Schleicher & Schuell). Primary antibodies were purchased from Mitoscience (MS105, MS203, MS304, MS404, MS502) except polyclonal antibodies against subunit *e* and *g* which were produced by Eurogentec. Rabbits were immunized with a mix of LKRIARELAEDDSIL and RYNYLKPRAEEERI peptides for subunit *e* and GEIIGKRGIIGYDV and VNSAQTGSFKQLTVK peptides for subunit *g*. Secondary antibodies were goat anti-rabbit and anti-mouse peroxidase-conjugated (Jackson ImmunoResearch). western-blots were revealed using the enhanced chemiluminescence method (Amersham Biosciences) on a CCD camera (GeneGnome, Syngene, Ozyme). After ECL detection, signal intensities were quantified with ImageJ [37].

### Transmission Electron Microscopy

Transmission electron microscopy analyses were done according to Eskelinen [38]. Briefly, adherent cells were fixed in 2% glutaraldehyde, 0.1 M HEPES pH 7.4 buffer followed by post-fixation with 2% reduced OsO_4_. Cells were further stained with 2% uranyl acetate, and submitted to dehydration in a graded series of ethanol. Cells were then embedded in Epoxy resin, and thin sections of 80 nm were cut on a microtome. The sections were finally stained with uranyl acetate and lead citrate for observation at 80 kV under a HITACHI H7650 transmission electron microscope (PIE Bordeaux Imaging Center Bordeaux Segalen University).

### Cellular O_2_ Flux Consumption Measurements

Oxygen consumption flux in HeLa cells was measured with a XF24 Extracellular Flux Analyzer (Seahorse Bioscience, Billerica, MA, USA). *Scramble* and *ShATP5I* transduced cells were seeded in XF 24-well cell culture microplates (Seahorse Bioscience) at 2×10^4^ cells per well in 500 µL of growth medium and incubated at 37°C under 5% CO_2_ for 24 h. Assays were initiated by replacing the initial growth medium with medium supplemented with OXPHOS substrates (10 mM malate, 10 mM pyruvate and 25 mM succinate). Cells were incubated at 37°C for 60 min and oxygen consumption were measured in the presence of OXPHOS substrates with/without addition of 0.5 µg/mL oligomycin or 0.6 µM CCCP (carbonylcyanide p-fluoro methoxyphenylhydrazone). Cells were counted after respiratory flux measurement for standardization. Statistical analysis were performed using Shapiro-Wilk and ANOVA 1 factor tests.

### Citrate Synthase Activity and Lactate Content Measurement

Activity of the mitochondrial matrix enzyme citrate synthase was assessed in cell homogenates to provide an estimate of the mitochondrial enzymatic content. Citrate synthase activity was measured according to the procedure previously described [39], with one unit of citrate synthase (E.C. 2.3.3.1 formerly E.C. 4.1.3.7) being considered equal to the reduction of 1 µmole of 5-5′-dithiobis-2-nitrobenzoic acid per min.

For the determination of lactate concentration in the medium, transduced cells were seeded in a 24-well microplate (2×10^4^ cells per well). The medium was taken every 10 hours and the cells were trypsinized and counted. The medium was incubated 8 min at 100°C. After a 8 min centrifugation at 4000×*g*, the supernatant was sampled and frozen at −80°C. For each sample, lactate quantity was enzymatically measured by a spectrophotometric determination [40].

### Fluorescence Microscopy

4×10^4^ cells were simultaneously spotted on glass bottom Petri dishes and transducted with lentiviral particles (*Scramble*, *ShATP5I* or *ShATP5L*). 72 hours after transduction, the medium was replaced and mitochondrial network morphology was observed in living adherent cells with a fluorescence microscope Olympus IX81 coupled to a thermostatic incubator (37°C, 5% CO_2_).

## Results

### Depletion of Subunits *e* and *g*

**Figure 1:**
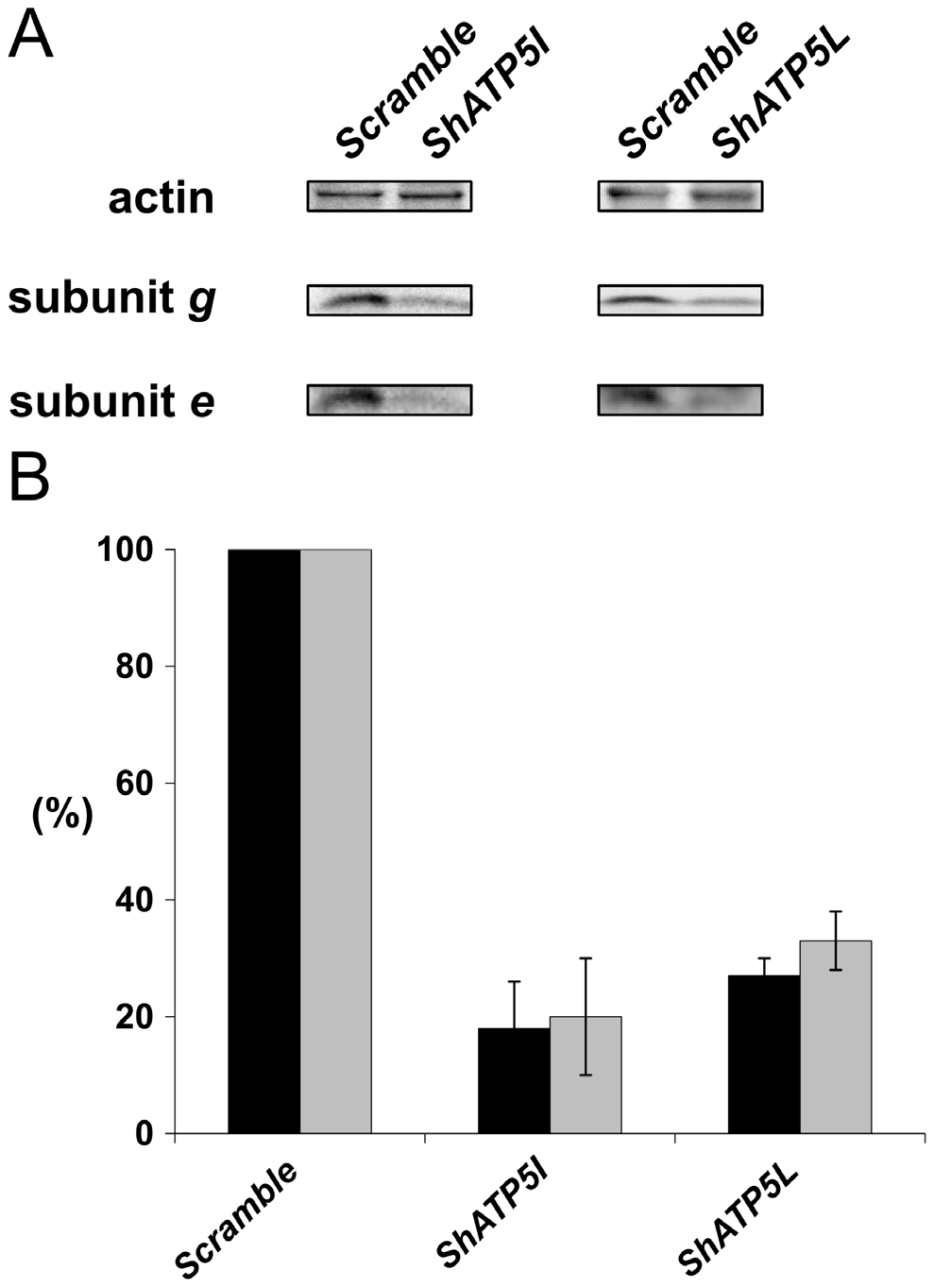
Subunits *e* and *g* depletion. (A) 72 h after transduction, total protein extracts (70 µg) were separated by Tris-tricine SDS- PAGE and transferred onto nitrocellulose membrane. The blots were probed with polyclonal antibodies raised against subunits *e*, *g* and actin as an internal standard (*Scramble*: control cells, *ShATP5I* and *ShATP5L*: cells transduced with shRNA against subunits *e* and *g*, respectivily). (B) Quantification of the signal intensity normalized with actin, expressed as a percentage of the control (black bar: subunit *e*; gray bar: subunit *g*; error bar: standard deviation). Percentages are the mean of 4 independent experiments.

To characterize the implication of subunits *e* and *g* in the function and the supramolecular organization of ATP synthase in mammalian cells, we used lentiviral particles to introduce shRNA sequences allowing the interference with the mRNA encoding subunits *e* or *g*. Quantification of the silencing efficiency relied on the use of antibodies directed towards the proteins of interest (Figure 1). The amount of subunit *e* was decreased by 82% ± 8 in *ShATP5I* transduced cells, indicating a high silencing efficiency, whereas the amount of subunit *g* was decreased by 67% ± 5 in *ShATP5L* transduced cells. These experiments allowed us to validate that *(i)* the antibodies were specific of subunits *e* and *g* and that *(ii)* transduction of shRNA by lentiviral particles led to a significant decrease in subunits *e* and *g* content in HeLa cells.

Previous work performed in yeast had demonstrated that the down-regulation of subunit *e* induces a decrease in the amount of subunit *g*, while the down-regulation of subunit *g* had no effect on subunit *e* accumulation, suggesting that only the assembly of subunit *g* in the yeast ATP synthase was dependent on the presence of the subunit *e* [15]. In HeLa cells, the attenuation of subunit *e* (*ShATP5I*) was correlated to a decrease in subunit *g* in the same proportion (82% ± 8 and 80% ± 10 respectively). The same observation was performed upon depletion of subunit *g* (S*hATP5L*), where a decrease in the amount of subunit *e* was observed in the same proportion (67% ± 5 and 73% ± 3 respectively) (Figure 1B). In HeLa cells, the absence of subunit *e* was correlated to the absence of subunit *g* and inversely. This result was the first tangible indication that the oligomerization process in mammalian cells may be different from yeast.

Since *ShATP5I* transduced cells presented a higher efficiency of subunits *e* and *g* depletion, we decided to use this shRNA for all the experiments presented in this work.

### Depletion of Subunits *e* and *g* Affects ATP Synthase Monomer Assembly and Destabilizes Oligomers

**Figure 2:**
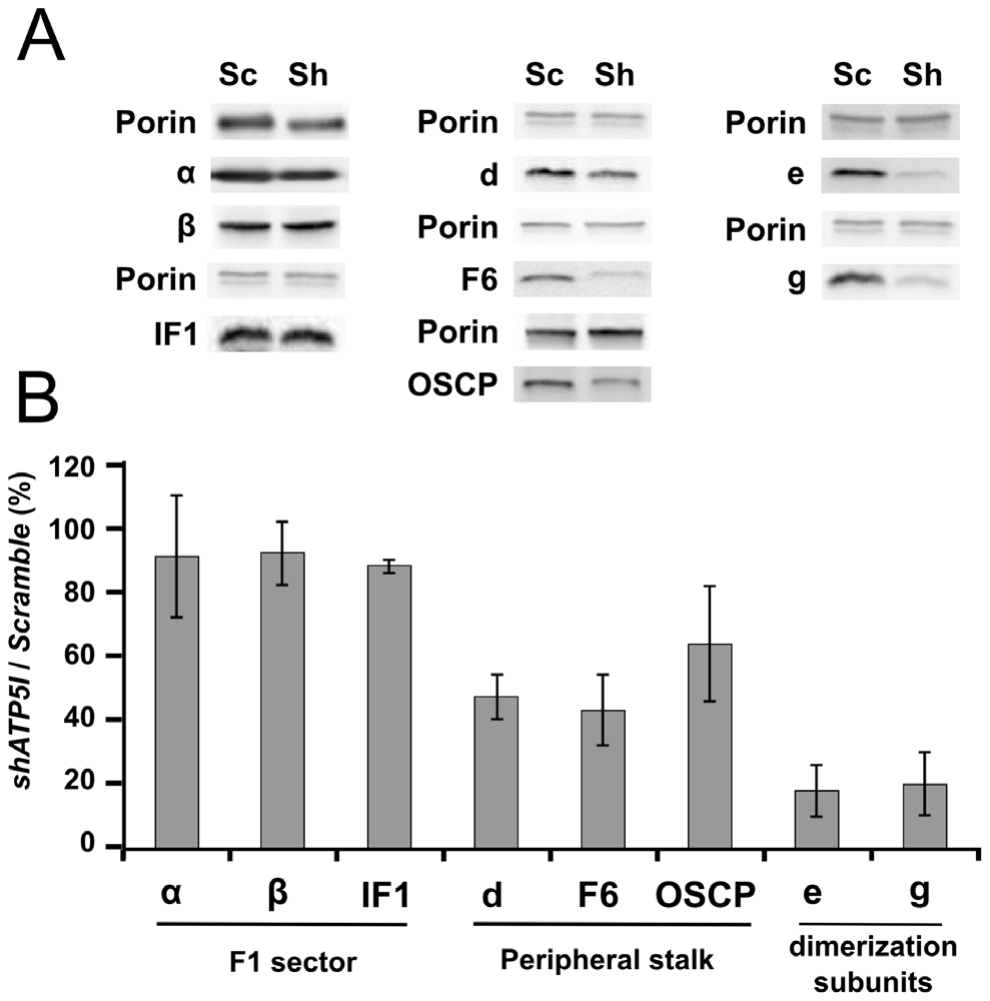
Steady state analysis of ATP synthase assembly in the absence of subunits *e* and *g*. (A) Mitochondrial proteins (50 µg) were separated by Tris-tricine SDS-PAGE. Subunits from the F_1_ and the F_0_ sectors were revealed by western blot using the appropriate antibodies (Sc: *Scramble*, Sh: *shATP5I*). (B) For each subunit, the signal was normalized to the signal of porin. Results obtained with *shATP5I* transduced cells were compared to control (*Scramble*) and are presented as a percentage. Measures are the mean of three different mitochondrial preparations (error bar: standard deviation).

Our primary aim was to characterize the consequences of subunits *e* and *g* depletion on ATP synthase assembly. We thus studied steady state accumulation of subunits which compose the F_1_ sector (*α*, *β*) and *IF1*, or the F_0_ sector (*d*, *F6*, *OSCP*) by western blot analyses (Figure 2).

In cells depleted in subunits e and g, contents of α and β (91% ± 20 and 92% ± 10 respectively) were not significantly different from *Scramble* control cells (Figure 2B). These results suggested that the accumulation of the subunits that compose the F1 sector was not modified by subunits e and g depletion. The content of the regulatory subunit IF1 was also almost identical to the control cells (88% ± 2). Surprisingly, accumulation of the subunits which compose the peripheral stalk was decreased (Figure 2B). Indeed, the relative amounts of subunits OSCP, d and F6 were reduced in mitochondria isolated from cells depleted in subunits e/g by 36%, 53% and 57% respectively. Based on these data, it appeared that the amount of correctly assembled mitochondrial ATP synthase (F1 and F0 sectors coupled) would be decreased by about 50% in mitochondria of *ShATP5I* transduced cells.

Compared to what was known from studies using yeast as a model [8], [15], this result was unexpected. This led us to investigate what the impact of subunits *e*/*g* depletion was on the supramolecular organization of ATP synthase. Mitochondrial supercomplexes from *Scramble* and *ShATP5I* transduced cells were solubilized with the indicated digitonin-protein ratios (g/g) and in-gel ATPase activity was revealed after protein complexes separation by BN-PAGE or CN-PAGE. Using increasing concentrations of digitonin allowed us not only to extract more proteins from the membranes but also to address the question of the stability of ATP synthase supercomplexes (dimers and oligomers). Indeed, higher digitonin-protein ratio are expected to destabilize oligomeric species to the profit of dimers and monomers of the wild type enzyme, whereas only monomers should be isolated regardless of the digitonin-protein ratio with dimerization/oligomerization impaired enzyme as previously described in yeast [8], [15].

**Figure 3:**
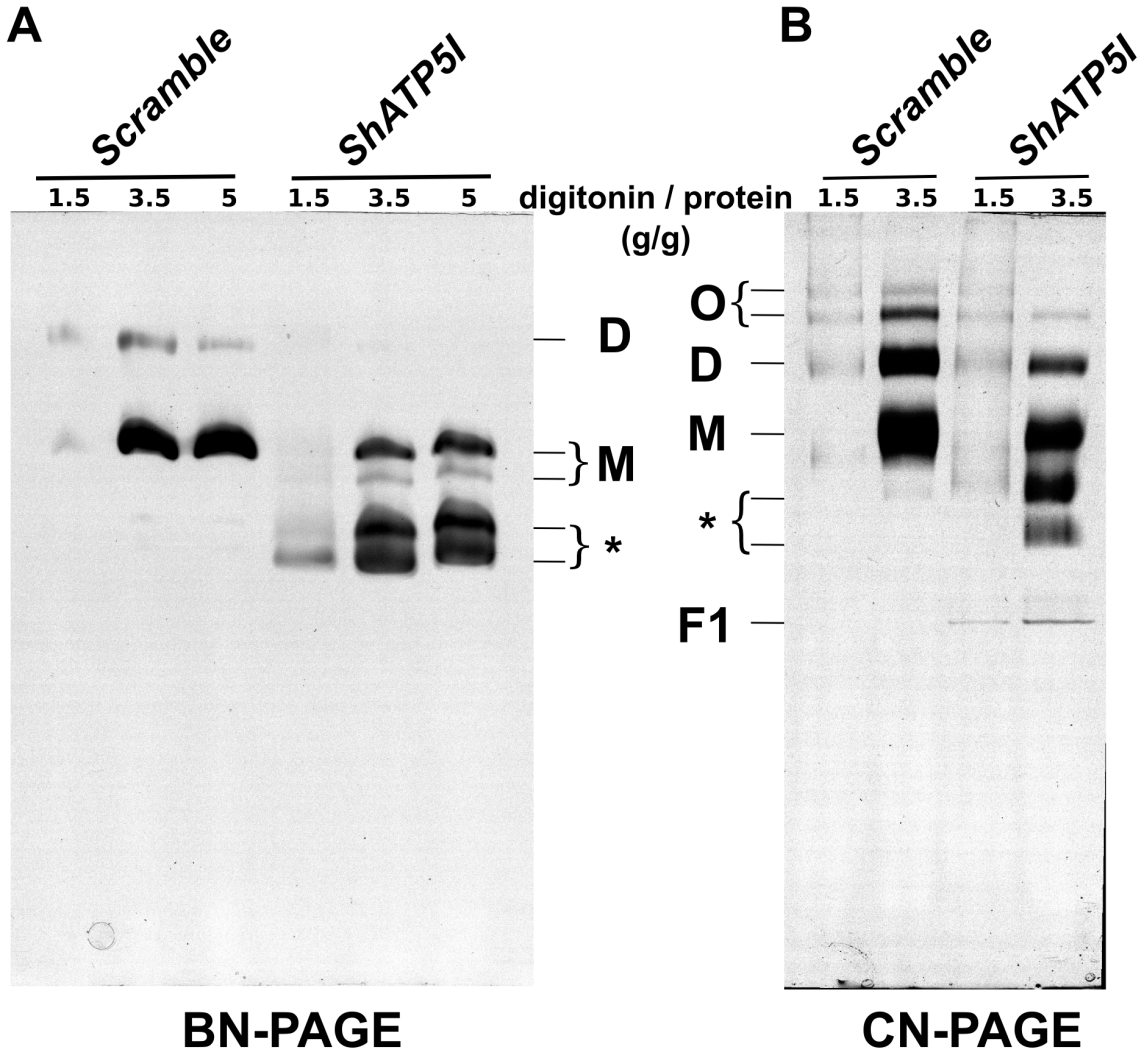
ATP synthase oligomers are destabilized after depletion of subunits *e* and *g*. *Scramble* and *ShATP5I* mitochondria were solubilized with the indicated digitonin-protein ratios (g/g). Mitochondrial complexes were separated by CN-PAGE or BN-PAGE and the gels were incubated with ATP-Mg^2+^ and Pb^2+^ to reveal the ATPase activity. (F1: F_1_ sector; M: monomer; D: dimer; O: oligomer; * : ATPase doublet). This figure in inverted colors is representative of five independent experiments.

For *Scramble* control mitochondria, in-gel ATPase activity revealed two bands in the BN-PAGE (Figure 3A). These bands were attributed to the monomeric and dimeric forms of ATP synthase as previously described [41]. The increase in digitonin-protein ratio was correlated to an increase in intensity of the monomers. However, it was not possible to observe oligomers at low digitonin-protein ratio with this technique. The reason why they could not be observed after BN-PAGE is unclear but in agreement with what was suggested by Wittig [42], Coomassie blue could destabilize the supramolecular organization of ATP synthase during electrophoresis. Thus, the oligomeric state of ATP synthase was assayed by CN-PAGE (Figure 3B). After in-gel activity staining, the presence of two more bands of high apparent molecular weight corresponding to oligomers of ATP synthase were revealed.

For *ShATP5I* attenuated mitochondria, analysis of digitonin extracts by BN-PAGE did not reveal the presence of ATPase dimers (Figure 3A). Instead, two bands at the apparent size of the monomeric enzyme were detected. In the CN-PAGE experiment, monomeric, dimeric and oligomeric species were observed (Figure 3B). Compared to *Scramble* control lanes, intensities of higher bands were clearly diminished with high digitonin-protein ratio (3.5 g/g) whereas they were similar at low digitonin-protein ratio (1.5 g/g, see Figure S2 for better visualization). The interpretation of the latter observation was not possible due to the very low amount of ATP synthase solubilized under this condition. However, our data at high digitonin-protein ratio suggested that depletion in subunits *e* and/or *g* destabilized ATP synthase oligomers. This point will be debated in the discussion section.

Our analysis of *ShATP5I* ATPase also revealed the presence of two bands at a lower apparent molecular weight, below the monomeric ATP synthase (Figure 3(*)). We can only speculate on the nature of this doublet detected when subunits *e* and *g* are down-regulated. It is noticeable that a faint ATPase activity was also present below the ATP synthase monomer in the CN-PAGE control lane at high digitonin-protein ratio. The fact that F_1_ ATPase activity [41] could be revealed in the *ShATP5I* lane, below the doublet in the CN-PAGE, suggested that they could be constituted by F_1_F_0_ ATP synthase lacking some subunits.

### Destabilization of ATP Synthase Oligomerization Affects Mitochondrial Network Morphology and Ultrastructure

**Figure 4:**
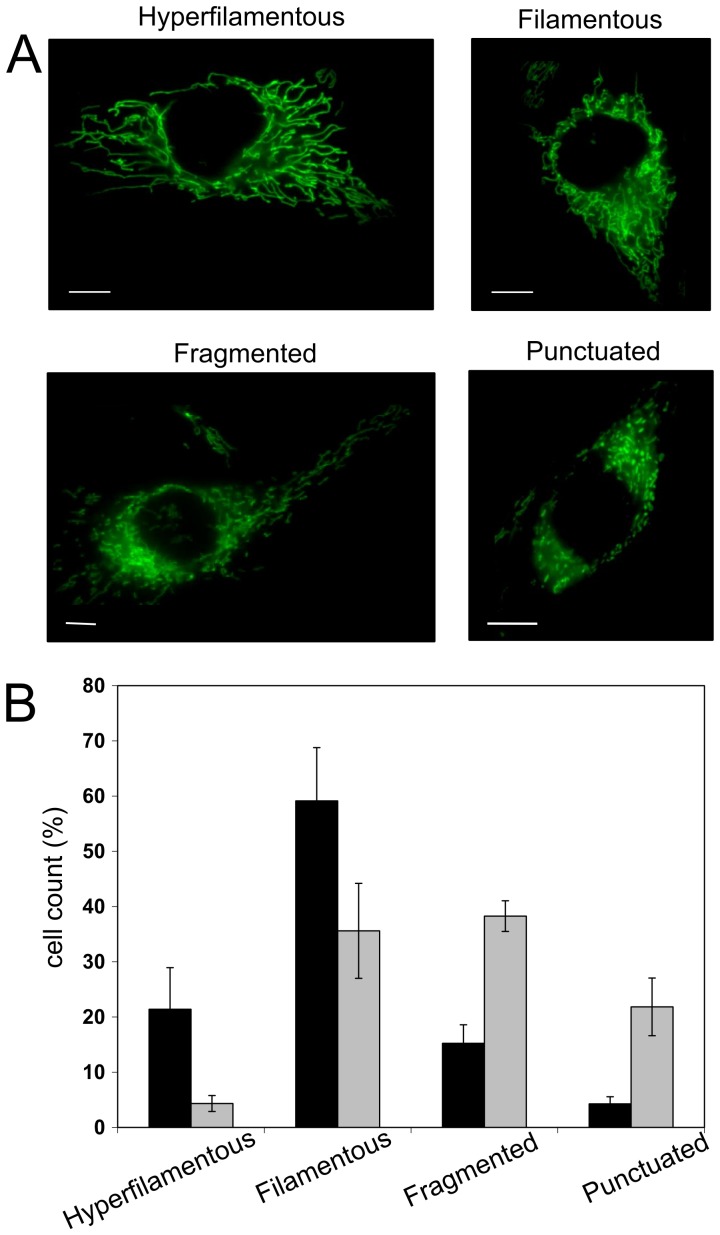
ATP synthase destabilization induces fragmentation of the mitochondrial network. HeLa cells were transduced with either the *Scramble* or *ShATP5I* lentiviral particles. 72 hours after transduction, mitochondrial network morphology was observed on adherent cells by fluorescence microscopy. (A) Representative morphologies of the mitochondrial network classes (bar = 10 µm). (B) Percentages of the different classes in the two cellular populations. Cells were manually classified by two different persons on 200 randomly selected cells. Counts are the mean of 4 independent experiments (black bar: *Scramble*; gray bar: *ShATP5I*; error bar: standard deviation).

We then assayed the mitochondrial network morphology in HeLa cells depleted or not in subunits *e* and *g* using a mitochondrial-targeted GFP. A statistical analysis of the mitochondrial network was performed. Network morphologies were classified into four different categories (Figure 4A). Hyperfilamentous morphology corresponded to cells exhibiting at least one mitochondrial tubule crossing the whole cell. Filamentous morphology was the classical morphology. Fragmented morphology was where tubules were shorter and punctuated morphology was where tubules were no longer visible but replaced by dots.

The statistical distribution of mitochondrial network morphologies in *Scramble* and *ShATP5I* transduced cells is presented in Figure 4B. In *Scramble* control conditions, 81% of the cells adopted filamentous and hyperfilamentous mitochondrial morphology. Fragmented and punctuated networks proportions were low (15 and 4% respectively). In *ShATP5I* transduced cells, mitochondrial organization was inverse: only 40% of the networks displayed a filamentous morphology (hyperfilamentous 4% and filamentous 36%) whereas 60% exhibited a fragmented or punctuated network (38 and 22 % respectively).

Thus, depletion in subunits *e* and *g* led to a mitochondrial network fragmentation. Because this depletion was also responsible for the destabilization of ATP synthase supramolecular forms, we investigated a potential effect on mitochondrial ultrastructure.

**Figure 5:**
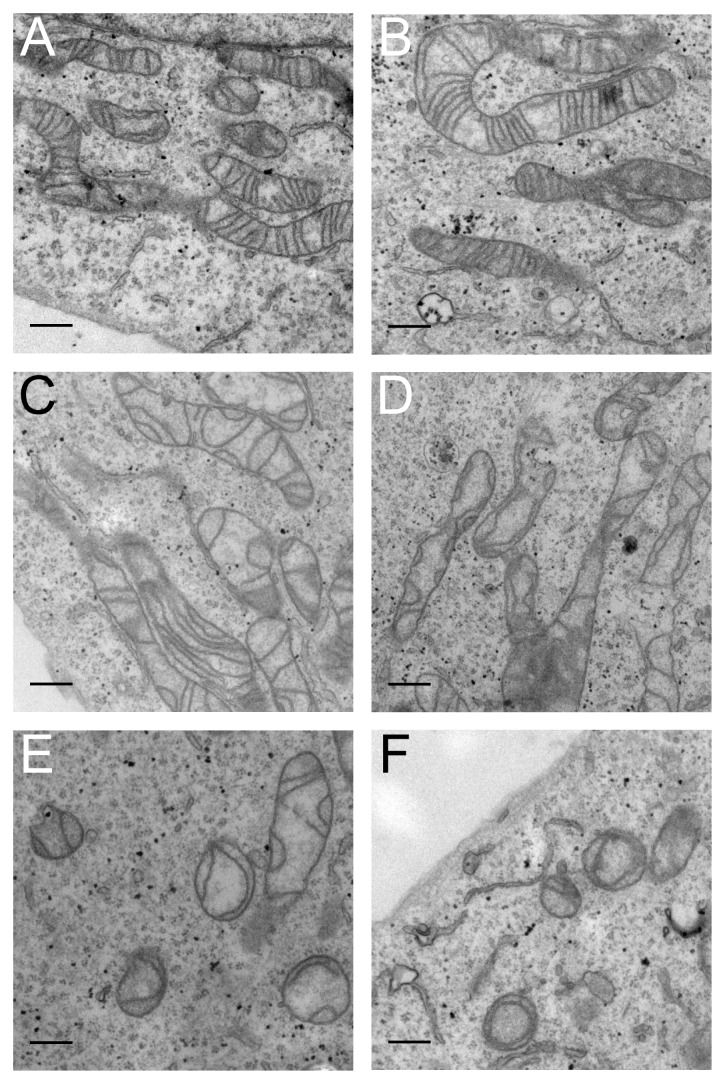
ATP synthase destabilization affects mitochondrial ultrastructure. 72 hours after transduction with *Scramble* (A,B) or *ShATP5I* (C,D,E,F) lentiviral particles, adherent cells were fixed and observed by electron microscopy as described in Materials and methods (bar = 0.5 µm).

Adherent *Scramble* and *ShATP5I* transduced cells were observed by transmission electron microscopy (Figure 5). Mitochondria from *Scramble* control cells displayed a canonical elongated or curved morphology with numerous transverse *cristae* (Figure 5A,B). A majority of mitochondria from *ShATP5I* transduced cells presented abnormal ultrastructures. Sections of mitochondria did not reveal the presence of canonical *cristae*. Instead, the inner membrane formed arch-like structures that seemed to partition the matrix, or protracted structures parallel to the longest side of mitochondrial section (Figure 5C,D). Electron micrographs also revealed the presence of smaller mitochondria, probably resulting from the network fragmentation, exhibiting the same abnormal organization of the *cristae*. Ultimately, circular sections where the inner membrane deformation led to horseshoe-shaped *cristae* were also observed (Figure 5E,F). As these experiments were performed on adherent cells, and these circular structures were visible on the periphery of the cells, we concluded that they were spherical mitochondria rather than transversal sections of elongated ones. These data were consistent with the mitochondrial network fragmentation of subunits *e*/*g* attenuated cells observed by fluorescence microscopy.

Altogether, these observations showed that subunits *e* and *g* attenuated cells presented mitochondrial ultrastructure alterations. This strongly suggests that in human cells, there is a correlation between depletion of subunits *e* and *g*, destabilization of ATP synthase supramolecular forms and modification of the *cristae* morphology.

### ATP Synthase Destabilization Affects the OXPHOS Pathway

We then assessed the consequences of ATP synthase destabilization on cell physiology by monitoring the outcome of this process on cell growth. Doubling-times determined by non linear regression from growth curves revealed that *ShATP5I* transduced cells double their population in 24 ± 1 hours, as compared to 20 ± 2 hours for *Scramble* control cells (Table 1). To investigate whether this doubling-time increase was due to a higher mortality of the *ShATP5I* transduced cells, viability was tested by propidium iodide exclusion measured by flow cytometry analysis (Figure S1). The proportion of cells labeled by propidium iodide was similar in *Scramble* and *ShATP5I* transduced cells (5.4% and 6.6% respectively). Therefore, no increase in cell death could be detected upon depletion of subunits *e* and *g*.

**Table 1:** Doubling-time and citrate synthase activity.

	Doubling-time(hours)	Citrate synthase activity	
		(pmol/min/mg)	(pmol/min/10^6^ cells)
***Scramble***	20 ± 2	51.4 ± 5.9	18.4 ± 3.0
***ShATP5I***	24 ± 1	49.7 ± 3.8	16.3 ± 2.2

Doubling-time was determined by non linear regression from growth curves. Citrate synthase activity was measured according to the procedure presented in Materials and methods. Values were normalized by the protein quantity (mg) or the cell number (10^6^). All the the values presented here are the mean of 3 independent experiments.

**Figure 6:**
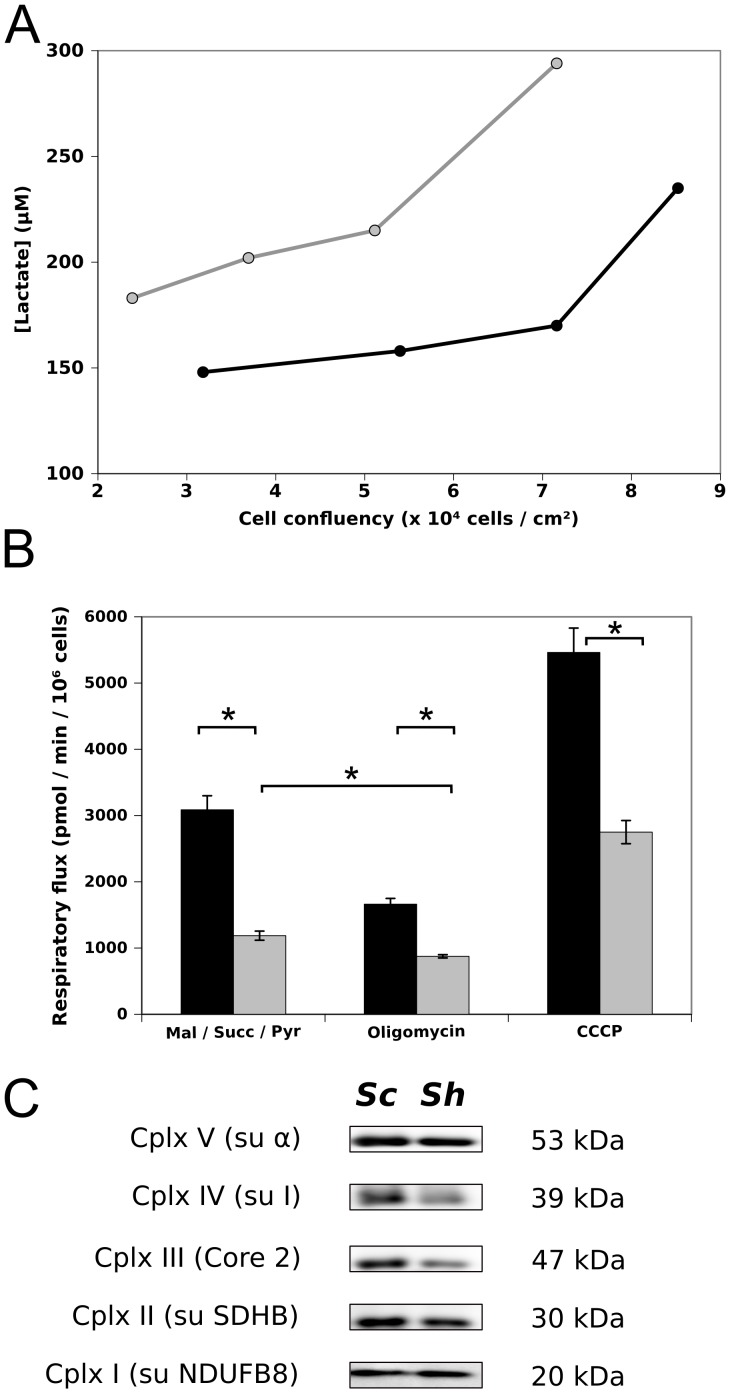
Functional consequences of the depletion of subunits *e* and *g*. (A) Extracellular lactate concentration (µM) released by *Scramble* (white dot) and *ShATP5I* (black dot) was measured during cell growth (representative of 3 independent experiments). (B) Oxygen consumption flux were performed as described in Materials and methods on adherent cells. O_2_ flux consumption were measured in the presence of malate (0.5 M), pyruvate (0.5 M) and succinate (0.5 M) supplemented with oligomycin (0.5 µg⋅mL^−1^) or CCCP (0.6 µM). Each measurement was performed three times in 5 minutes. Data are the mean of 4 experiments and are expressed as picomole of O_2_ per minute per 10^6^ cells (black bar: *Scramble*; gray bar: *ShATP5I*; error bar: standard deviation; *: P<0.002). (C) Mitochondrial proteins (50 µg) were separated by Tris-glycine SDS-PAGE. Subunits representative for each respiratory complex were revealed by western blot using the appropriate antibodies (*Sc*: *Scramble*; *Sh; ShATP5I*). This figure is representative of three independent experiments.

Depletion in subunits *e* and *g* led to a rapid acidification of the culture medium which prompted us to assess the aftermath of their attenuation on cell metabolism. Measurement of lactate concentration in the medium revealed a huge increase in the secretion of this metabolite by *ShATP5I* transduced cells. This difference reached 80% at 7.2×10^4^ cells / cm^2^ which is close to confluence (Figure 6A). As this finding could reflect an increase in the glycolytic pathway at the expense of the respiratory pathway, we next investigated the consequences of subunits *e*/*g* depletion on the OXPHOS. Respiratory flux measurements were done on adherent cells in the presence of respiratory substrates (malate, succinate, pyruvate) and specific inhibitor of ATP synthase (oligomycin) or uncoupler (CCCP) (Figure 6B). Basal respiratory flux rates, that represent a phosphorylating state, were reduced by 61% in subunit *e*/*g* attenuated cells compared to control (1187 ± 69 and 3087 ± 212 pmol O_2_ / min / 10^6^ cells respectively). Similarly, respiratory rates measured in the presence of CCCP, corresponding to the maximum capacity of the respiratory chain, were decreased by 49% in cells attenuated in subunits *e* and *g* compared to control cells (2750 ± 175 and 5462 ± 368 pmol O_2_ / min / 10^6^ cells respectively). Residual respiration rate in presence of oligomycin was also higher in *Scramble* cells than in *ShATP5I* transduced cells (1662 ± 87 and 875 ± 25 pmol O_2_ / min / 10^6^ cells respectively). The oligomycin insensitive respiration was reduced compared to the basal respiration in both cases, suggesting that in the *ShATP5I* cells, the ATP synthase was functional under phosphorylating state.

This clearly demonstrated that the attenuation of the expression of subunits *e* and *g* in HeLa cells drastically diminished the OXPHOS pathway activity. We envisaged and tested different hypotheses that could explain the reduction of the respiratory flux observed in these experiments. Cells depleted in subunits *e*/*g* could have: (i) a lower mitochondrial content compare to control cells, (ii) a decrease in the OXPHOS complexes activity related or not to (iii) a reduced content of some respiratory chain complexes and/or ATP synthase.

### ATP Synthase Destabilization is Related to a Decrease in Complexes III and IV Content

In mammalian cells, measuring citrate synthase activity is considered to give an accurate estimation of the cellular mitochondrial content. Measurements were performed as described in Materials and methods and the results obtained are presented in Table 1. Citrate synthase activities were not significantly different between *Scramble* and *ShATP5I* transduced cells. This result argued in favor of a similar mitochondrial enzymatic content in both transduced cells. Mitochondrial respiratory flux alteration in cells depleted in subunits *e* and *g* was therefore not due to a decrease in the cellular mitochondrial content.

To further evaluate whether the respiratory chain enzymatic equipment was similar between *Scramble* and *ShATP5I* transduced cells, mitochondria were isolated from both cellular populations. Mitochondrial proteins were separated by SDS-PAGE and the amount of one reporter subunit for each respiratory complex was assessed by western-blot (Figure 6C). The mitochondrial content of complexes I and II did not vary between the two cellular populations whereas the content of complexes III and IV was decreased in subunits *e*/*g* depleted cells (Figure 6C). This decrease in complexes III and IV content was consistent with the lowered respiratory flux in *ShATP5I* transduced cells, and in agreement with the decrease in the oxygen consumption rate assessed in the presence of uncoupler.

## Discussion

Our main goal was to evaluate the influence of the ATP synthase oligomerization process on mitochondrial ultrastructure and cell physiology in mammalian cells. To address this question we used a shRNA strategy to down-regulate the expression of subunits *e* and *g* in HeLa cells, as these two ATP synthase subunits were proposed to be involved in the dimerization and oligomerization processes by analogy to their yeast counterparts.

During the last ten years, various topological models of the membrane proteins involved in yeast ATP synthase oligomerization were proposed. They all take into account the fact that this oligomerization implies the existence of two interfaces (see [3] for review). The first interface would be mediated by homo-interactions between subunits *e* and *g* and the second one, involving subunits *4* and *h* (*b* and *F_6_* in mammals respectively) would be stabilized by the presence of the very same subunits *e* and *g*. In yeast, deletion or attenuation of the expression of one or the other of those subunits results in a complete loss of oligomeric ATP synthase, as assayed by native electrophoresis and suggested by electron microscopy [43]. In mammalian cells, the role of subunits *e* and *g* in ATP synthase dimerization/oligomerization process was a subject of speculations. In this paper we show that human subunits *e* and *g* are involved in the oligomerization of mitochondrial ATP synthase as depletion of those subunits decreased the amount of dimers and oligomers to the benefit of monomers in native electrophoresis.

In the yeast *S. cerevisiae*, subunits *e* and *g* are usually considered accessory subunits as their loss has no effect on the function, the assembly or the stability of the ATP synthase monomer. In light of our work, in mammalian cells, the situation is totally different. In cells with down-regulated subunits *e* and *g*, a fraction of the ATP synthase was destabilized on native electrophoresis, its ATPase activity being revealed between the monomer and the free F_1_. Such a migration profile has already been observed in human ρ^0^ cells where subunits *a* and *A6L* are missing in the ATP synthase and was attributed to the accumulation of an assembly intermediate [41]. In our case studying the subunit composition of this band by classical 2D approach was not possible due to the number of proteins co-migrating with this complex and to the absence of an exhaustive set of antibodies directed toward ATP synthase subunits (see Figure S3 for more information). Here, the ATPase activity of these intermediate bands increases with detergent-protein ratio (CN-PAGE) or in the presence of Coomassie blue (BN-PAGE). We construe this result as an indication that the enzyme would lose some of the F_0_ subunits during detergent extraction and/or migration and we surmise that this intermediate ATPase activity is the result of the destabilization of ATP synthase monomers. This *in vitro* destabilization is correlated with a steady state 50% decrease in the amount of F_0_ subunits *d*, *F6* and *OSCP* while the amount of F_1_ subunits (α and β) and of *IF1* remained constant in attenuated cells. Whether the latter point is the consequence of the ATP synthase destabilization observed in BN/CN PAGE remains an open question. Previous studies demonstrate that subunits *e* and *g* are strongly associated with the monomer purified from beef heart mitochondria [44] while they are lost in the monomer purified from yeast [6], [45]. Thus, in human cells, the absence of interactions between the ATP synthase monomer and subunits e and/or g would either impair the proper assembly of the enzyme or cause proteolysis of the destabilized enzymes.

The main phenotype observed in yeast when ATP synthase oligomerization is impaired is a complete loss of mitochondrial structure [8]. Human cells depleted in subunits *e* and *g* display a mitochondrial alteration as the network was fragmented, but the observed ultrastructure is different from that depicted in yeast. We did not observe any of the onion like structures that were previously described. Instead, we observed a disorganization of the inner membrane forming arch-like or longitudinal *cristae* in the mitochondria. Various cell treatments leading to the loss of OXPHOS complexes and impairment of the inner membrane potential are known to promote the fission of the mitochondrion and alter the ultrastructure of the organelle. For instance ρ^0^ cells (treated with ethidium bromide) or chloramphenicol treated cells (inhibiting mitochondrial protein synthesis) possess a fragmented mitochondrial network and exhibit a few abnormal *cristae* (chloramphenicol treated cells) or no *cristae* at all (ρ^0^ cells) [46]. Moreover, the impairment of the fusion machinery in knockouts of both alleles of *mfn1* or *opa1* results in the fragmentation of the mitochondrial network, but in that case *cristae* are still visible and do not exhibit any of the phenotype we describe here [47]. Thus it is very unlikely that the fragmentation and *cristae* reorganization observed in the present work result from a bioenergetics defect or an impairment of the fusion machinery. The shRNA approach used in this work allowed us to down-regulate the expression of subunits *e* and *g* by 80%. In these conditions, we were able to detect the presence of oligomeric ATP synthase by native electrophoresis. This “mild” disorganization of the *cristae* is similar to the phenotype of a partial down-regulation of the expression of subunits *e* and *g* in yeast, where trabecular *cristae* were observed [15]. We may speculate that in HeLa cells, the oligomeric enzyme with a sub-stoichiometric subunits *e* and *g* content is no longer able to curve the membrane properly, leading to this intermediate morphology.

The absence of subunits *e* and *g* in yeast has a very minor impact on the OXPHOS activity. Indeed, no effect could be observed on the O_2_ flux consumption of isolated mitochondria although the *in vitro* maximum ATPase activity of the F_1_F_0_ complex was reduced [15]. Our work shows that despite a similar mitochondrial content, HeLa cells depleted in subunits *e* and *g* have a 50 % decrease in their respiratory rate accompanied by an increase in the lactate released in the medium which reflects an increase in glycolysis. This has to be correlated with a 50% diminution of the active F_1_F_0_ ATP synthase and the diminution of the steady-state quantities of core 2 and of the subunit 1 of complexes III and IV respectively. In the yeast models of NARP and MILS syndromes, where the gene encoding subunit *6* is mutated, a linear correlation between ATP synthase and complex IV activity has been shown, caused by a decrease in the steady-state amount of Cox1p [48], [49]. To our knowledge, it is the first time that a relationship between the impairment of ATP synthase and the respiratory chain activity is described in a non cybrid mammalian cell model. Complex V deficiency usually implies mitochondrial genes encoding subunits *6* and *8* or nuclear genes *ATP12* and *TMEM70* encoding chaperones involved in ATP synthase assembly [25]. Further investigations are necessary to fully characterize the consequences of subunits *e* and *g* attenuation on complexes III and IV and particularly to determine how the assembly *versus* degradation balance is impaired and leads to the metabolic reorientation we observed in this work.

A recent publication suggests that the ATP synthase dimer would be involved in the permeabilization of the mitochondrial inner membrane. This permeability transition pore is a key effector of cell death and it would be composed of the ATP synthase dimer which is able to mimic the electrophysiological behavior of this pore when inserted in artificial membrane bilayer [50]. Although this finding needs confirmation, the tools developed in our study may be useful to precise and or confirm the nature of this pore.

In conclusion, we demonstrate for the first time, that in a human cell line, subunits *e* and *g* are involved in ATP synthase dimerization/oligomerization processes. These subunits are necessary for the stability of the monomeric as well as the dimeric and oligomeric enzyme. Nevertheless we can not exclude that other proteins like *IF1* may be involved in this process. The down-regulation of both subunits *e* and *g* and consequently the destabilization of ATP synthase has strong functional consequences. We propose that mutations or deletion in the genes encoding subunits *e* and/or *g* may have physiopathological consequences in multicellular organisms and has to be investigated.

## Supporting Information

Figure S1 (PDF)

Figure S2 (PDF)

Figure S3 (PDF)
