# Supplementary material for: Human F1F0 ATP Synthase, Mitochondrial Ultrastructure and OXPHOS Impairment: A (Super-)Complex Matter?
Source: PLoS One. 2013 Oct 2;8(10):e75429. doi: 10.1371/journal.pone.0075429 (PMC3788808; doi:10.1371/journal.pone.0075429)
Supplement: Figure S1 — (PDF) [file pone.0075429.s001.pdf]

Figure S1

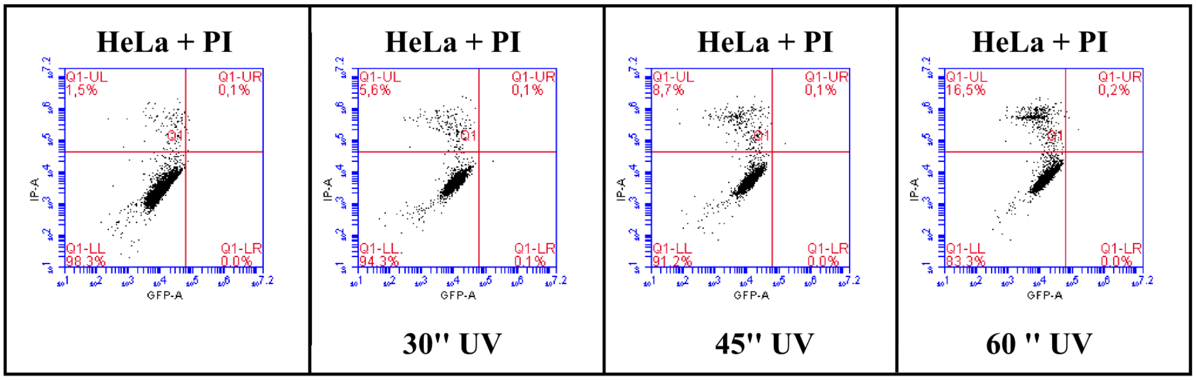

A

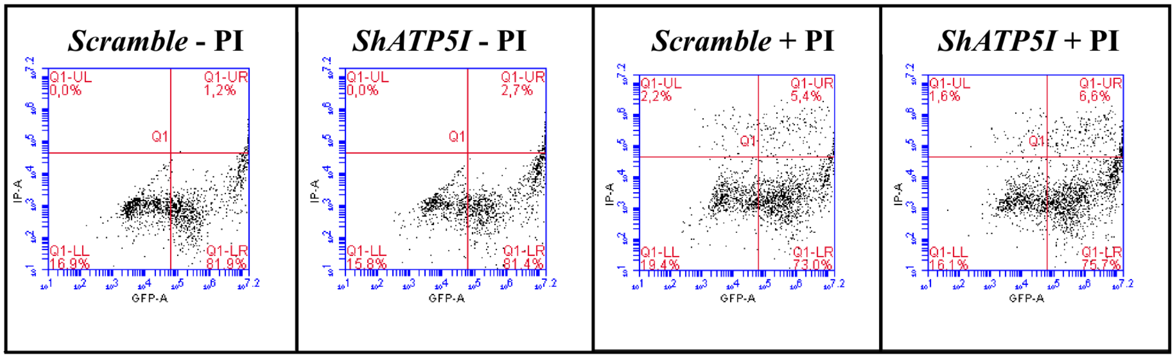

B

Cell viability assay. 72 hours after transduction, 3×10^4 cells, were filed in wells of a 24-well dish. After 24 hours of growth, cells were detached, rinsed and resuspended in 200 µL of DPBS. After addition of 0.5 µg of propidium iodide (PI), cells were incubated for 15 min at 37 ° C in the dark. The proportion of cells having fixed PI was determined by flow cytometry using a BD Accuri C6 Flow Cytometer. (A) non transduced cells were UV irradiated (45mJ/cm^2) during the indicated time five hours before PI treatment. (B) *Scramble* and *shATP5I* transduced cells treated or not with PI. This figure is representative of 3 independent experiments.
