# Supplementary material for: Human F1F0 ATP Synthase, Mitochondrial Ultrastructure and OXPHOS Impairment: A (Super-)Complex Matter?
Source: PLoS One. 2013 Oct 2;8(10):e75429. doi: 10.1371/journal.pone.0075429 (PMC3788808; doi:10.1371/journal.pone.0075429)
Supplement: Figure S2 — (PDF) [file pone.0075429.s002.pdf]

Figure S2

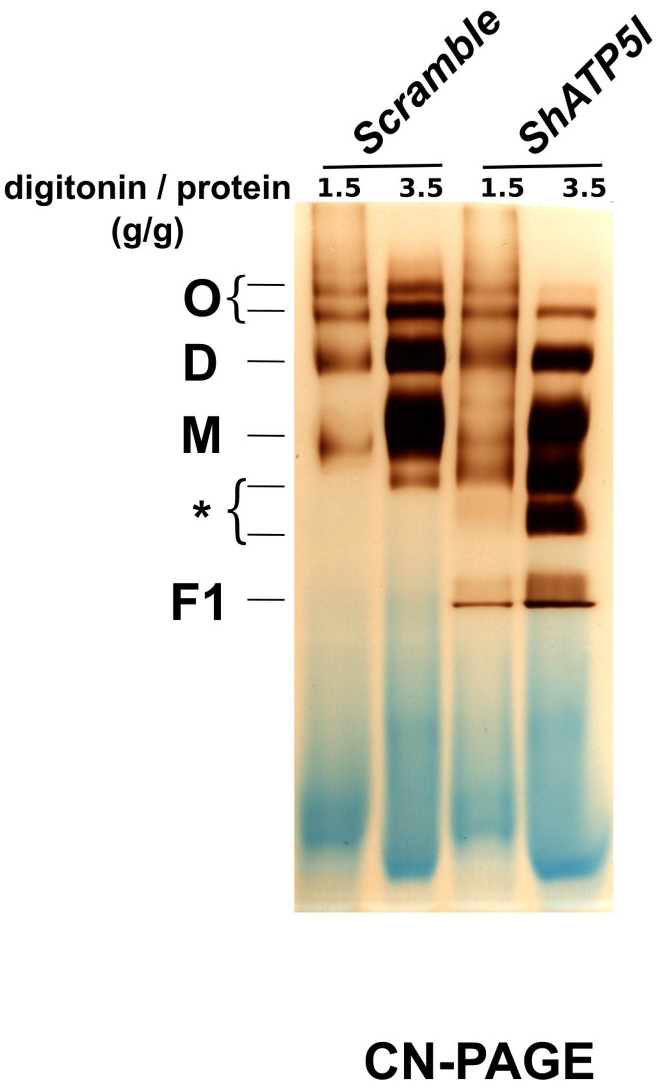

High sensitivity ATPase activity detection (cf Figure 3, right panel):

Scramble and *ShATP5I* mitochondria were solubilized with the indicated digitonin-protein ratios (g/g). Mitochondrial complexes were separated by CN-PAGE and the gels were incubated with ATP-Mg^{2+} and Pb^{2+} to reveal the ATPase activity. The signal was enhanced with an ammonium sulfide treatment as described by Suhai *et al* (2009). F1: F_1 sector; M: monomer; D: dimer; O: oligomer; * : ATPase doublet.

Suhai T, Heidrich NG, Dencher NA, Seelert H (2009) Highly sensitive detection of ATPase activity in native gels. Electrophoresis 30: 3622–3625.
