# Supplementary material for: Human F1F0 ATP Synthase, Mitochondrial Ultrastructure and OXPHOS Impairment: A (Super-)Complex Matter?
Source: PLoS One. 2013 Oct 2;8(10):e75429. doi: 10.1371/journal.pone.0075429 (PMC3788808; doi:10.1371/journal.pone.0075429)
Supplement: Figure S3 — (PDF) [file pone.0075429.s003.pdf]

Figure S3

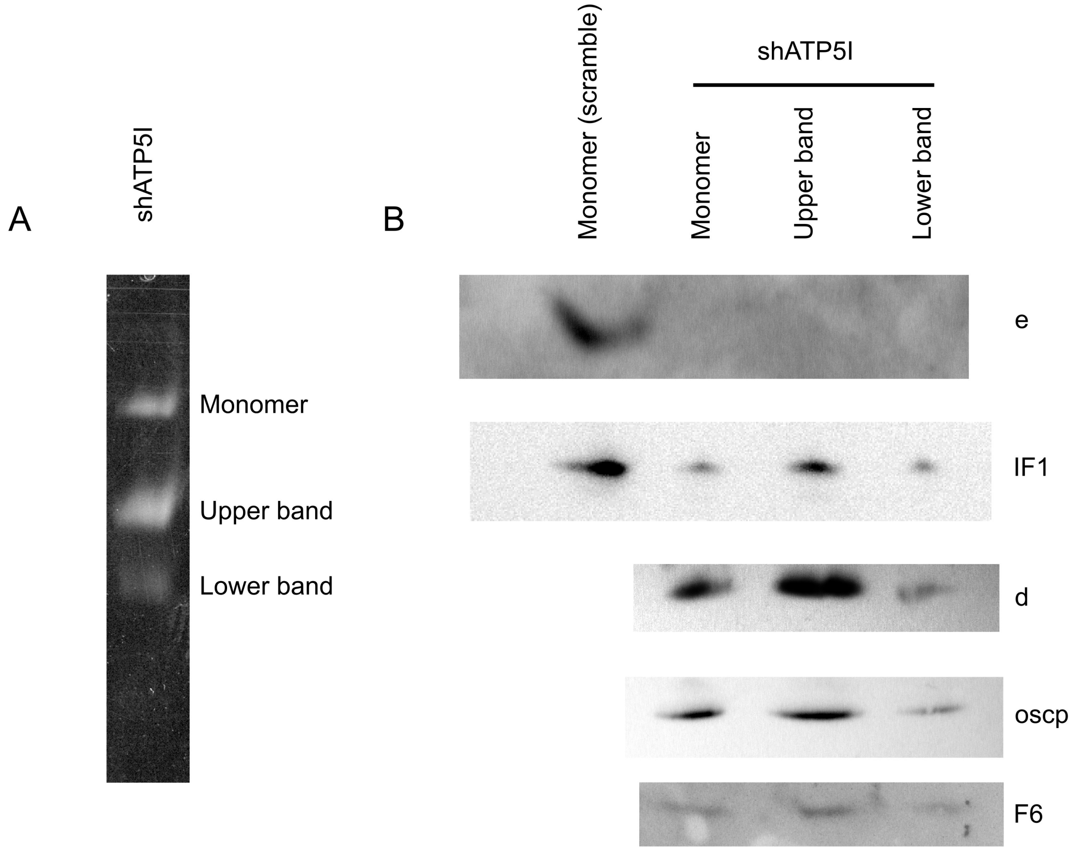

2D electrophoresis ATP synthase characterization was performed by cutting monomer, upper and lower band from a CN PAGE experiment (3.5 g digitonin / g protein) and submit them to SDS PAGE. The indicated subunits were revealed by western blot with the appropriate antibodies.

In gel ATPase activity and western blot suggest that these ATP synthase complexes are at least composed of the F_1 moiety (subunits α, β, γ, δ and ε) and some of the subunits from the peripheral stalk (d, oscp, F6). We may also speculate that subunit b is present as it is needed for subunits d, oscp and F6 anchorage to the complex.
